# Statistical Analysis of nnU-Net Models for Lung Nodule Segmentation

**DOI:** 10.3390/jpm14101016

**Published:** 2024-09-24

**Authors:** Alejandro Jerónimo, Olga Valenzuela, Ignacio Rojas

**Affiliations:** 1 Computer Engineering, Automatics and Robotics Department, University of Granada, 18071 Granada, Spain; irojas@ugr.es; 2 Department of Applied Mathematics, University of Granada, 18071 Granada, Spain; olgavc@ugr.es

**Keywords:** statistical analysis, computed tomography, lung nodule, lung cancer, deep learning, segmentation

## Abstract

This paper aims to conduct a statistical analysis of different components of nnU-Net models to build an optimal pipeline for lung nodule segmentation in computed tomography images (CT scan). This study focuses on semantic segmentation of lung nodules, using the UniToChest dataset. Our approach is based on the nnU-Net framework and is designed to configure a whole segmentation pipeline, thereby avoiding many complex design choices, such as data properties and architecture configuration. Although these framework results provide a good starting point, many configurations in this problem can be optimized. In this study, we tested two U-Net-based architectures, using different preprocessing techniques, and we modified the existing hyperparameters provided by nnU-Net. To study the impact of different settings on model segmentation accuracy, we conducted an analysis of variance (ANOVA) statistical analysis. The factors studied included the datasets according to nodule diameter size, model, preprocessing, polynomial learning rate scheduler, and number of epochs. The results of the ANOVA analysis revealed significant differences in the datasets, models, and preprocessing.

## 1. Introduction

Lung cancer is a significant health problem, being the leading cause of cancer-related deaths worldwide. The latest GLOBOCAN 2022 [1] estimates the incidence and mortality of different types of cancer and is produced by the International Agency for Research on Cancer (IARC): lung cancer accounts for more than 1.8 million deaths (18.7% of all cancer types). In 2024, 611,720 people will die of cancer, of whom 125,070 (20,44%) will die of lung cancer, in the United States [2]. In Europe, the situation does not improve [3]. Lung cancer remains the first cause of cancer-related deaths among men, with 153,032 predicted deaths. For women, the predicted mortality is 84,402 compared with the 76,041 deaths observed in 2018. The prediction for 2050 indicates that the number of diagnosed cases will increase. Therefore, it is crucial to use early diagnostic methods to reduce disease prognosis and improve patients’ quality of life.

Lung cancer is diagnosed through physical examination, biopsy, or imaging, using tools such as magnetic resonance imaging (MRI) and computed tomography (CT) [4]. A pulmonary nodule is an abnormal area of the lung. Pulmonary nodules are common findings, detected in approximately 30% of chest CT scans and 1.6 million patients annually in the US. They are categorized as small solid (<8 mm), larger solid (≥8 mm), and subsolid, which includes ground-glass and part-solid nodules [5]. At least 95% of all pulmonary nodules identified are benign, but the risk of malignant tumors increases with nodule size, from <1% for nodules <6 mm to 64–82% for nodules >20 mm [5,6]. Nodules >10 mm are considered large nodules, while nodules <3 mm are micronodules [7]. Other risk factors include patient age, smoking history, and nodule characteristics, such as irregular borders and growth rate [8,9].

Lung nodule segmentation solutions can be divided into two categories: traditional segmentation and deep learning methods. Morphological operations, active contours, and region-growing are common traditional segmentation methods [10,11,12]. Although traditional methods are resource-efficient, deep learning techniques provide superior results. Thanks to deep learning techniques, it is possible to extract relevant features that perform pixel-by-pixel classification in an image, thereby allowing for more precise segmentation. These techniques can be applied to different types of images, such as CT and histopathological images [13,14]. In recent years, autoencoder architectures like U-Net have been used to solve this problem, specifically in medical imaging. With the use of these segmentation models, a radiologist can detect nodules that might otherwise go unnoticed, assist in the final diagnosis, and even study changes in nodule size over time. Lung nodule segmentation in CT images remains a challenging task, due to the variety of nodule shapes, sizes, and densities, as well as their similarity to surrounding structures. First, large databases with nodules of different characteristics are required. Due to the sensitive nature of such images, public databases are scarce. The most commonly used public databases are LIDC-IDRI [15] and LUNA16 [16]. The segmentation model requires precise annotations from experts, which is time-consuming. These segmentation masks must be labeled correctly, to indicate the exact shape of the nodule for generalization to the learning process. Recent innovations include multi-crop CNNs [17], dual-branch networks [18,19], and region-based fast marching methods [20]. Although these techniques have had promising results, challenges remain in achieving high sensitivity with low false-positive rates, managing different types of nodules, and developing robust models applicable to diverse patient databases [21]. Another important aspect to highlight is the training pipeline. In most segmentation problems, it is necessary to manually optimize and configure the entire process, including preprocessing, normalization, hyperparameters, and architecture configuration. In addition, this process must be performed for each dataset. To address these issues, the nnU-Net framework [22] was published in 2021 by Isensee et al. With nnU-Net, it is not necessary to manually configure and adjust the entire pipeline. Thus, different components of the pipeline, such as normalization, hyperparameters, and architecture configuration, adapt according to the properties of the dataset and image modality.

The nnU-Net framework automates configuring and training CNNs for medical image segmentation via a systematic approach. It begins with dataset feature extraction, known as data fingerprint, where the image modality and intensity value distribution are analyzed. Based on this data fingerprint, rule-based parameters are established, such as normalization tailored to each image modality. In CT scans, global z-score normalization is applied. The framework then automatically determines the network topology and batch size, based on the available GPU memory. The loss function and key hyperparameters, like learning rate, number of epochs, and optimizer, are fixed. Finally, the network is trained using the previous configuration.

The use of nnU-Net provides a good starting point for lung nodule segmentation. Although the initial results are acceptable, the framework does not account for different types of relevant processing for the problem and different hyperparameter values that can improve the segmentation model’s accuracy.

For this reason, the objective of this paper was to conduct a statistical analysis to study the influence of applying different processing techniques, models, hyperparameters, and types of nodules, so as to achieve the best approach to solving the problem. For this purpose, we present an ANOVA (analysis of variance) analysis, to study the impact of the aforementioned factors on the segmentation model’s accuracy and training time. During this study, we tested two different architectures based on U-Net, two different datasets of large and small nodules, employing different preprocessing techniques, such as contrast enhancement and lung segmentation, and different hyperparameter values, such as the polynomial learning rate scheduler and the number of epochs.

## 2. Materials and Methods

### 2.1. Architectures and Related Works

Among the most widely used architectures for lung nodule segmentation, U-Net [23] stands out for achieving good results in medical image segmentation problems via the use of skip connections. However, the base U-Net model fails to extract sufficient features for precise segmentation. To address these issues, several modifications to the architecture and the use of different preprocessing techniques have been proposed. Chaudhry et al. [24] proposed a 2D base model of U-Net and used transfer learning through pre-training on the LIDC-IDRI dataset [15] to segment nodules in the UniToChest dataset, achieving good results. On the other hand, we have also observed the use of residual blocks in the literature, to prevent relevant information loss, and the use of Atrous convolution [25,26] to obtain multiscale features. In this way, nodules of different sizes can be detected [27,28]. In 2020, Zhou et al. [29] proposed the U-Net++ architecture, which adds more depth levels to the U-Net architecture and skips connections between different levels. In addition, they added deep supervision, allowing work at different image scales and improving results. Isensee et al. [22] proposed the nnU-Net framework, where they focused on optimizing the deep learning pipeline rather than modifying the architecture. Therefore, they proposed several 2D and 3D U-Net base architectures for segmentation problems of all kinds of medical images.

In the literature, review articles have conducted meta-analyses of different deep learning techniques for lung screening and diagnosis, addressing classification and segmentation problems, and analyzing their impact on various metrics [30,31]. Regarding similar works that perform statistical analysis, there are few proposals in the literature that exhaustively analyze different types of processing, hyperparameters, and architectures applied to this specific problem using statistical tests, although there are studies, such as that by Fusco et al. [32], which have conducted a chi-square test to examine significant differences between chest X-ray images and CT scans in the context of machine learning and deep learning methods applied to COVID-19. Chen et al. [33] conducted a comparative study of various processing techniques on the LIDC-IDRI dataset [15] and different architectures. The authors investigated the impact of two preprocessing techniques: cropping, for extracting the region of interest (ROI), and lung parenchyma segmentation. In addition, they analyzed the impact on eight segmentation models. The authors analyzed the average performance of the different models and the execution time. However, they did not use statistical tests like ANOVA to determine if there were significant differences between the different techniques employed. This paper makes a novel contribution by performing ANOVA statistical analysis in the field of nodule segmentation by examining different techniques and deep learning methods.

### 2.2. Data Resource

**Figure 1:**
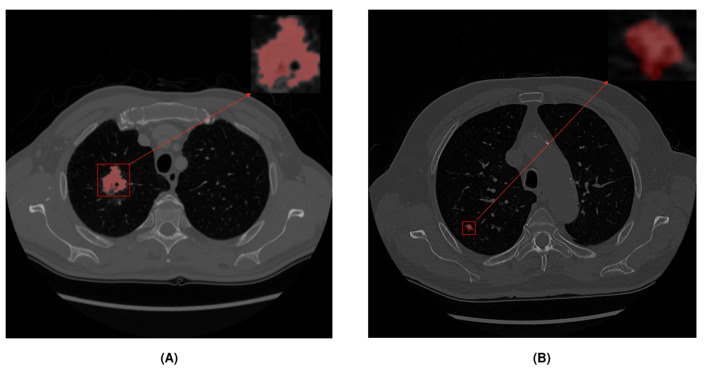
Different subsets according to nodule diameter size. Red color represents the nodule area: (**A**) Example of a large nodule (>10 mm). (**B**) Example of a small nodule (<10 mm).

The UniToChest dataset [24] consists of about 300 k CT scans of pulmonary nodules. This is the largest publicly available lung nodule dataset. Images were acquired in the DICOM format, and each scan with nodules included an image and mask, both of 512×512 size. For slices without nodules, there was no mask. The proposed dataset also contains a wide variety of images of different sizes compared to other public datasets. The nodule diameter range is between 1 and 136 mm. During this study, we used 22,713 CT scans with nodules. The dataset includes division of images into training, validation, and test sets. To study the influence of different nodule sizes, we created two subsets from the original dataset, while respecting the original training, validation, and test sets. These two nodule subsets corresponded to larger nodules that were more likely to be malignant (greater than 10 mm) and smaller nodules (less than 10 mm) that could develop cancer in the future. Figure 1 shows an example of a CT scan for each subset. Table 1 shows all the image divisions made and the number of images per split.

### 2.3. Models and Preprocessing

Throughout this study, several models provided by the nnU-Net framework were used, to study their influence on performance and time. The nnU-Net framework offers a series of models based on the U-Net architecture that are tailored to the type of image processing. The framework includes a 2D model based on the classic implementation, a 3D model designed for low-resolution 3D images, and a cascade model for high-resolution images. For this study, we used only the 2D model. Additionally, the latest update of the framework includes three models that use residual blocks in the encoder, aiming to preserve more information along with the skip connections already present in the U-Net architecture. With the use of residual blocks, segmentation accuracy could be improved because we retained information that might be lost across the encoder layers. These three models were adjusted to the capacity of the available GPU. In this study, the *ResEncUNetM* architecture [34] was evaluated. Table 2 shows the configuration of various hyperparameters and the loss function used in the experiments. During the experimental phase, we used the fixed values provided by nnU-Net and modified the polynomial learning rate scheduler and the number of epochs.

**Figure 2:**
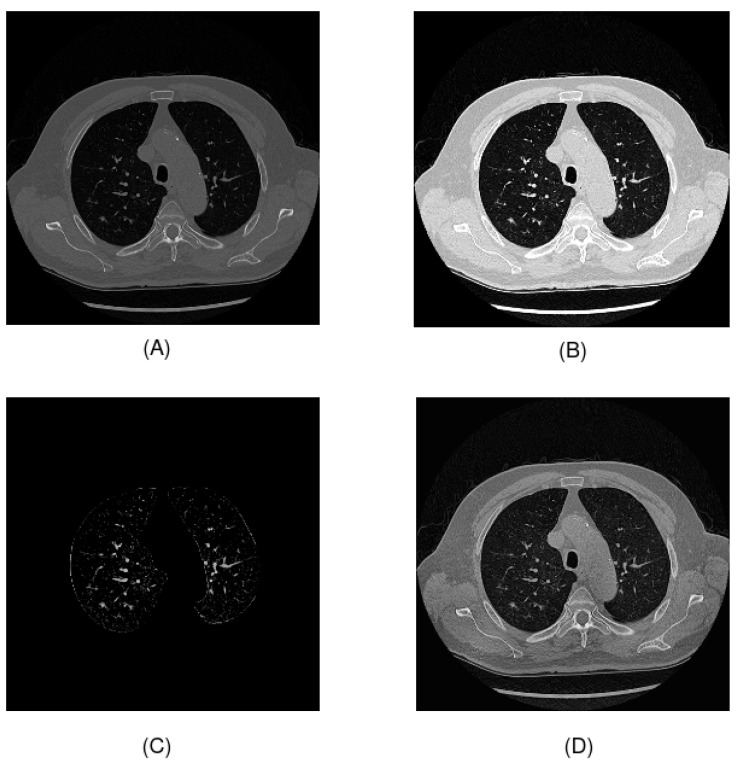
Preprocessing techniques used in this study: (**A**) Hounsfield units preprocessing; (**B**) windowing; (**C**) U-Net R231 and thresholding; (**D**) CLAHE.

Regarding preprocessing, numerous techniques are available for working with CT scans. In this study, we used the two preprocessing techniques for Hounsfield units and contrast enhancement proposed in the original article by Chaudhry et al. [24] and two proposed techniques based on lung segmentation and thresholding and on contrast-limited adaptive histogram equalization (CLAHE) for contrast enhancement. The application of these four techniques on a CT image is shown in Figure 2:

First, we applied preprocessing of the Hounsfield units. The raw pixel values of DICOM images are scanner values ranging from 0 to 4095. These values need to be transformed into Hounsfield units for clinical interpretation and to construct the final image. In some images, there exists a value of −2000, which is outside the scanner value range. For this reason, we removed the noise in the transformation by changing the value −2000 to 0, which is the minimum value. In addition, we applied windowing, to enhance the contrast of the image. This technique is widely used for working with CT scans. In this study, we followed the preprocessing proposed by Chaudhry et al., setting a window width of 1600 and a window center of −500. The selection of these values was made to detect the Hounsfield unit values that were of interest to the problem. In this case, the value of −500 HU represented lung tissue, and all values outside the range defined by the window width and window center were converted to black pixels if they were below the lower limit and to white pixels if they were above the upper limit. Another technique used to improve contrast is contrast-limited adaptive histogram equalization (CLAHE). Using this method, the local contrast of an image is enhanced in small areas and noise is reduced compared to other contrast-equalization techniques. The main parameters of this method are the clip limit, which is the maximum value of the histogram in a region, and the tile grid size, which defines the size of the local regions to which histogram equalization is applied. In this study, we set the clip limit to 2 and the tile grid size to 8×8. Finally, we applied another preprocessing proposal consisting of lung area segmentation and thresholding. The objective of this preprocessing was to reduce the amount of irrelevant information in the tomography scan as much as possible, highlighting the nodules. To perform segmentation, the U-Net R231 model was used [35]. To further reduce the information, grayscale pixels were removed by setting a threshold of 35.

### 2.4. Statistical Analysis

Analysis of variance (ANOVA) is a robust statistical tool used to determine if there are significant differences between the means of multiple samples. In the context of deep learning models, the ANOVA test can be used to analyze how variation in different parameters affects model performance, such as segmentation accuracy and execution time. In this study, we used the following methodology:

1. **Parameter and Level Definitions:** The first step involved selecting the parameters of the deep learning model to be analyzed and defining different levels for them. We selected the following ones:
  - **Dataset:** We used two different datasets with large and small nodules to study the impact of nodule diameter size in the model (see Table 3).
  - **Model:** We used two different models provided by nnU-Net to study the impact of using residual connections in the encoder of U-Net (see Table 4). The first model was a classic U-Net implementation, and the second was a modified encoder using residual connections like ResNet architecture, in addition to the skip connections of U-Net.
  - **Preprocessing:** In the literature, many preprocessing techniques have been applied to CT scans. In this study, we used windowing, which is one of the most common techniques to enhance contrast (see Table 5). We preprocessed Hounsfield unit values by removing noise elements, following the original preprocessing of the UniToChest paper, and we evaluated two proposed preprocessing techniques, which consisted of segmenting the lung area, using the U-Net R231 model and thresholding to highlight nodules, and using CLAHE for contrast enhancement.
  - **Polynomial learning rate scheduler:** This is a technique to reduce the learning rate gradually. The scheduler depends on three factors: initial learning rate, number of epochs, and power. The polynomial learning rate scheduler follows the following equation:
    (1) $$\eta_{t}=\eta_{0}{\left(1-\frac{t}{T}\right)}^{p}$$
    where $\eta_{t}$ is the learning rate at epoch *t*, $\eta_{0}$ is the initial learning rate, *T* is the total number of epochs, and *p* is the power of the polynomial.
    A smaller exponent causes the learning rate to decay more slowly at the beginning of training and decay more rapidly at the end. However, a larger exponent will cause the learning rate to decay faster at the beginning and slowly at the end. During this analysis, we focused on the exponent rather than the initial learning rate, which was fixed at 0.01. We evaluated different exponent values, as shown in Table 6, including the recommended value provided by nnU-Net (0.90).
  - **Epochs:** Number of epochs during training. We evaluated different numbers of epochs to study the impact on model performance and training time, as illustrated in Table 7.
  - **Model Training and Data Collection:** We trained a model for each combination of parameters. For each run, we stored the results of the segmentation metrics that evaluated the performance of the model and the time to obtain it. This generated a tabular dataset, where each column represented the results of a specific parameter value and each row corresponded to an experiment. A total of 144 experiments were performed, using all possible combinations. For this analysis, we used the mean dice score coefficient (DSC) of the images in the test subset. DSC is one of the most common metrics used in lung nodule segmentation, and it measures the similarity between two masks.
  - **ANOVA Analysis:** ANOVA consists of comparing between-group and within-group variability.
    - **Null hypothesis** ($H_{0}$): The means of the segmentation metrics for different parameter values are equal.
    - **Alternative hypothesis** ($H_{1}$): At least a mean of the segmentation metric is different.
    We calculated the F statistic to compare between-group and within-group variability. If the F value was significantly large then the null hypothesis was rejected.
    If the null hypothesis was rejected, it could be concluded that the variation in the parameter significantly affected the accuracy of the model. Otherwise, we could not conclude that parameter had a significant impact.
    In this paper, we performed two-way ANOVA, a statistical method used to examine the influence of two independent categorical variables on one continuous dependent variable. This helped us to understand not only the individual effects of each factor but also how they worked together, providing a comprehensive view of the influences on the dice score metric and training time.

## 3. Results and Discussion

### 3.1. Two-Way Analysis of Variance for Dice Score

The ANOVA table (Table 8) breaks down the variability of DSC into contributions from various factors. Since the Type III sum of squares was chosen, the contribution of each factor was measured after removing the effects of the other factors; the *p*-values tested the statistical significance of each factor. Because seven *p*-values were less than 0.05, these factors had a statistically significant effect on the DSC at the 95.0% confidence level.

The results in Table 8 indicate that the three statistically significant factors were the dataset used, the model, preprocessing, and the number of epochs. Additionally, three of these factors had a *p*-value of 0, indicating that the null hypothesis could be rejected with high probability. When studying the interaction between two factors, we found that the dataset with preprocessing, the model with preprocessing, and the model with the number of epochs were statistically significant. To further analyze these variables, we conducted multiple comparisons to determine which means were statistically different.

**Multiple Range Tests for DSC by Dataset**

In Table 9, two homogeneous groups are identified according to the letters of the columns. There were no statistically significant differences between levels that share the same column of letters. The method currently used to discriminate between means is Fisher’s least significant difference (LSD) procedure. With this method, there is a 5.0% risk of stating that each pair of means is significantly different when the actual difference is equal to 0. Table 10 shows the estimated differences between each pair of means. An asterisk is placed next to one pair, indicating that the pair demonstrates statistically significant differences at the 95.0% confidence level.

**Figure 3:**
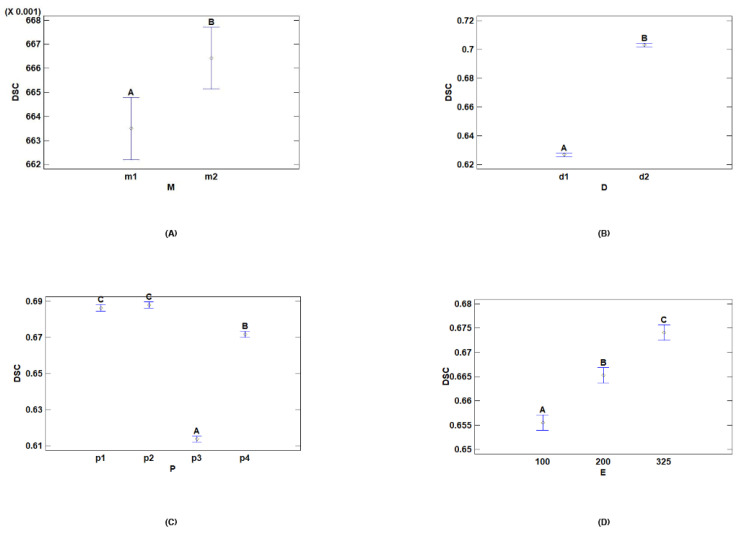
Means and 95.0 percent LSD intervals for DSC: (**A**) means and intervals for dataset; (**B**) means and intervals for model; (**C**) means and intervals for preprocessing; (**D**) means and intervals for epochs.

The previous results indicated that the nodule size significantly affected segmentation accuracy. Nodules with a diameter equal to or greater than 10 mm were easier to detect because they occupied a notable size, allowing the model to better extract size and shape features. Nodules smaller than 10 mm occupied less space compared to the total image size, making it more difficult to extract features, which decreased segmentation accuracy (Figure 3):

**Multiple Range Tests for DSC by Model**

Table 11 and Table 12 show that there were a total of two homogeneous groups, indicating significant differences between both models, notably influencing segmentation accuracy. In Figure 3, the U-Net model with residual components obtained better results than the base model. The use of residual components in the encoder preserved more information that could be lost with the base model across layers, improving the results.

**Multiple Range Tests for DSC by Preprocessing**

In Table 13, three homogeneous groups were identified according to the letters of the columns. There were no statistically significant differences between the Hounsfield units and windowing preprocessing because they belonged to the same homogeneous group. In Table 14, the asterisk next to five pairs indicates that these pairs demonstrated statistically significant differences at the 95.0% confidence level.

The above results show that preprocessing with Hounsfield units and windowing did not significantly differ. This means that compared with using the original Hounsfield values, using CT scans with more contrast did not influence the segmentation accuracy. However, windowing generally performed better, on average (Figure 3).

On the other hand, there were significant differences when lung segmentation preprocessing and thresholding were used, compared to other techniques. The same applied to the CLAHE technique. Figure 3 shows that these two techniques performed worse, on average, especially in the case of *p3*. By focusing on the lung area and minimizing image information, the model failed to correctly detect nodules in the dataset images.

**Multiple Range Tests for DSC by Epochs**

Table 15 and Table 16 show significant statistical differences between the different epoch levels. There were three distinct homogeneous groups corresponding to the three epoch levels. Figure 3 shows the differences between the levels: the higher the number of epochs, the greater the segmentation accuracy.

**Interactions with DSC**

**Figure 4:**
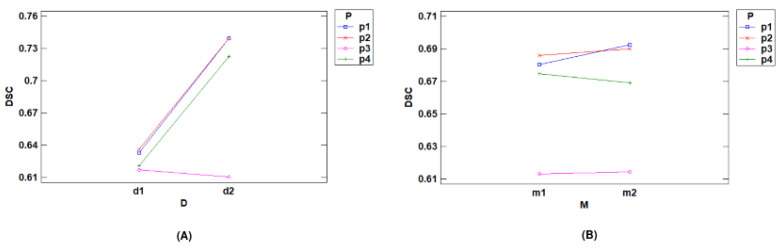
Interaction plots for DSC: (**A**) interaction plot between preprocessing and DSC for dataset; (**B**) interaction plot between preprocessing and DSC for model.

Figure 4 shows the most significant interaction plots for the DSC metric. Figure 4A shows the interaction between processing techniques and datasets. It can be observed that for the large nodule dataset, there was an increase in the DSC metric across most preprocessing techniques, except lung segmentation (*p3*). The Hounsfield units (*p1*) and windowing (*p2*) techniques obtained the highest DSC values and achieved better results than the other techniques. These results indicate that images with larger nodules tend to have better performance.

Figure 4B shows the interaction plot between the preprocessing techniques and the models. We can see that, once again, the *p1* and *p2* techniques achieved better results. Additionally, *p1* and *p2* showed an increase in DSC when moving from the baseline model (*m1*) to the residual model (*m2*). These results demonstrate that both preprocessing methods improved performance in terms of DSC when used with *m2*. In the case of *p3*, there was hardly any change when transitioning from one model to another. Finally, when using the CLAHE technique (*p4*) the results decreased as we switched from one model to another.

### 3.2. Two-Way Analysis of Variance over Time

As with the dice score analysis, Table 17 decomposes the variability of time (s) into contributions due to various factors. Since Type III sums of squares were chosen, and the contribution of each factor was measured after removing the effects of all the other factors, the *p*-values tested the statistical significance of each factor. Six *p*-values were less than 0.05; these factors had a statistically significant effect on t (s) at the 95.0% confidence level. The factors that significantly influenced training time were the model, preprocessing, and the number of epochs. When studying the interaction between two factors, we found that the model with preprocessing, the model with epochs, and the exponent with epochs were statistically significant. Additionally, all *p*-values were zero, indicating that the null hypothesis could be rejected with high probability. The remaining factors did not show statistically significant differences and did not influence time.

In the following subsections, the significant differences of the variables are discussed, with the exception of the number of epochs, as a higher number of epochs always consumes more time.

**Multiple Range Tests for Time (s) by Model**

**Figure 5:**
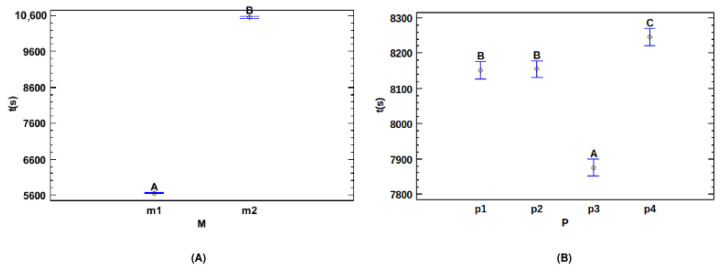
Means and 95.0 percent LSD intervals for time: (**A**) means and intervals for model; (**B**) means and intervals for preprocessing.

Table 18 and Table 19 show that there were quite significant differences between the two models, in terms of their influence on time. Both models formed different homogeneous groups, demonstrating their statistical differences. The base model was considerably faster than the residual model (Figure 5). Adding new residual layers increased the number of parameters in the network, making the training slower.

**Multiple Range Tests for Time (s) by Preprocessing**

For preprocessing, there were three homogeneous groups (Table 20). The preprocessing of Hounsfield units (*p1*) and windowing (*p2*) belonged to the same homogeneous group and did not significantly influence time. In contrast, Table 21 shows that lung segmentation (*p3*) and CLAHE (*p4*) had significant differences from the other preprocessing techniques, with *p3* being the fastest technique. Applying U-Net R231 and thresholding reduced training time because of the greater number of zero pixels compared to other techniques, which optimized hardware usage during training (Figure 5).

**Interactions with Time (s)**

**Figure 6:**
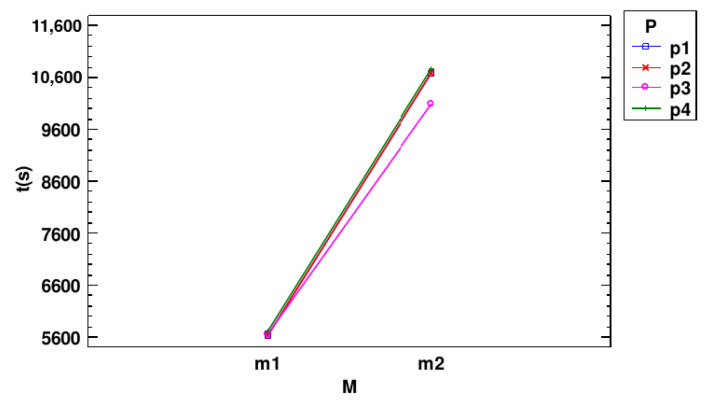
Interaction plot between model and preprocessing for time.

Figure 6 shows the interaction plot that illustrates how the two deep learning models performed relative to execution time when applying different types of preprocessing methods. The interaction plot reveals that the execution times associated with preprocessing methods *p1*, *p2*, and *p4* are nearly indistinguishable across models U-Net (*m1*) and residual U-Net (*m2*), as evidenced by their nearly overlapping lines. This indicates that selecting these preprocessing methods had a minimal impact on model performance, in terms of execution time. However, *p3* displays a notable deviation from this pattern, showing a distinct interaction with the models, where the change in execution time from *m1* to *m2* differed. In addition, *p3* was faster than the other preprocessing methods in the *m2* model.

## 4. Conclusions

This study performed an in-depth statistical analysis to assess the performance of the nnU-Net models for lung nodule segmentation, with an emphasis on varying preprocessing techniques and model configurations. The results demonstrate that both the preprocessing methods and model configurations significantly affected segmentation accuracy and training time.

Lung segmentation using preprocessing U-Net R231 and the thresholding technique resulted in the fastest processing times. In contrast, CLAHE, Hounsfield units preprocessing, windowing, and other preprocessing methods demonstrated significant differences in computational efficiency, highlighting the critical role of preprocessing in optimizing performance. Windowing was the preprocessing technique that achieved the best results, on average; however, it was not significantly different from preprocessing the images with the original Hounsfield values.

The basic U-Net model consistently outperformed the residual model, in terms of training time, which was confirmed by multiple range tests. However, the residual model achieved higher segmentation accuracy, particularly for nodules larger than 10 mm, which are most relevant to clinical practice. This indicates a necessary trade-off between computational efficiency and segmentation accuracy, depending on specific application needs.

The results demonstrate that varying the exponent values in the polynomial learning rate scheduler did not result in significant differences, thereby not affecting segmentation accuracy or time. However, the interaction between the chosen dataset and preprocessing and the model with preprocessing significantly influenced the segmentation results. In addition, the model and preprocessing factors, along with the number of epochs, had a notable impact on training time.

Two-way ANOVA revealed that the choice of model, preprocessing technique, and number of epochs significantly affected both the dice score and training time. These findings are essential for optimizing nnU-Net pipelines and enhancing the efficiency and accuracy of lung nodule segmentation.

## Figures and Tables

**Table 1:** UniToChest dataset splits across experiments.

	Original Splits	Big Nodules (>10 mm)	Small Nodules (<10 mm)
Train	18,534	11,445	7089
Validation	1712	1132	580
Test	2467	1514	953
Total	22,713	14,091	8622

**Table 2:** Below, nnU-Net fixed configuration in all experiments.

nnU-Net Configuration	
Loss function	Dice and cross-entropy
Optimizer	SGD with Nesterov momentum (μ = 0.99)
Initial learning rate	0.01
Data augmentation	Rotations, scaling, Gaussian noise, Gaussian blur, brightness, contrast, simulation of low resolution, gamma correction and mirroring

**Table 3:** Levels of dataset variable.

Dataset (D)	
**Level**	**Description**
d1	Small nodules subset
d2	Big nodules subset

**Table 4:** Levels of model variable.

Model (M)	
**Level**	**Description**
m1	Basic U-Net
m2	ResEncUNetM

**Table 5:** Levels of preprocessing variable.

Preprocessing (P)	
**Level**	**Description**
p1	Hounsfield units preprocessing
p2	Windowing preprocessing
p3	U-Net R231 lung segmentation + thresholding
p4	Contrast enhancement using CLAHE

**Table 6:** Levels of polynomial learning rate scheduler variable.

Polynomial Learning Rate Scheduler (Pl)	
**Level**	**Description**
pl1	0.50
pl2	0.75
pl3	0.90

**Table 7:** Levels of epochs variable.

Epochs (E)	
**Level**	**Description**
e1	100
e2	200
e3	325

**Table 8:** Two-way analysis of variance for DSC—Type III sum of squares.

Source	Sum of Squares	Df	Mean Square	F-Ratio	*p*-Value
**Main Effects**					
A: Dataset	0.4175	1	0.4175	3356.80	**0.0000**
B: Model	0.000614836	1	0.000614836	4.94	**0.0271**
C: Preprocessing	0.263374	3	0.0877914	705.86	**0.0000**
D: Polynomial Scheduler	0.000128853	2	0.0000644265	0.52	0.5964
E: Epochs	0.0165057	2	0.00825287	66.36	**0.0000**
**Interactions**					
AB	0.000404227	1	0.000404227	3.25	0.0726
AC	0.165121	3	0.0550402	442.54	**0.0000**
AD	0.0000382051	2	0.0000191025	0.15	0.8577
AE	0.00219469	2	0.00109734	8.82	0.0002
BC	0.00300852	3	0.00100284	8.06	**0.0000**
BD	0.000734474	2	0.000367237	2.95	0.0540
BE	0.00094172	2	0.00047086	3.79	**0.0240**
CD	0.000573798	6	0.000095633	0.77	0.5950
CE	0.00108091	6	0.000180152	1.45	0.1967
DE	0.000321692	4	0.000080423	0.65	0.6298
**Residual**	0.0307205	247	0.000124374		
**Total (Corrected)**	0.903263	287			

**Table 9:** Means and 95.0 percent LSD intervals for DSC by dataset.

D	Cases	Mean LS	Sigma LS	Homogeneous Groups
d1	144	0.626883	0.00092936	A
d2	144	0.703032	0.00092936	B

**Table 10:** Contrast comparison by dataset.

Contrast	Significant	Difference	+/− Limits
d1–d2	*	−0.0761486	0.0025887

* Asterisk denotes a statistically significant difference.

**Table 11:** Means and 95.0 percent LSD intervals for DSC by model.

M	Cases	Mean LS	Sigma LS	Homogeneous Groups
m1	144	0.663497	0.00092936	A
m2	144	0.666419	0.00092936	B

**Table 12:** Contrast comparison by model.

Contrast	Significant	Difference	+/− Limits
m1–m2	*	−0.00292222	0.0025887

* Asterisk denotes a statistically significant difference.

**Table 13:** Means and 95.0 percent LSD intervals for DSC by preprocessing.

P	Cases	LS Mean	LS Sigma	Homogeneous Groups
p3	72	0.613729	0.00131431	A
p4	72	0.671787	0.00131431	B
p1	72	0.686335	0.00131431	C
p2	72	0.687979	0.00131431	C

**Table 14:** Contrast comparison by preprocessing.

Contrast	Significant	Difference	+/− Limits
p1–p2		−0.00164444	0.00366097
p1–p3	*	0.0726056	0.00366097
p1–p4	*	0.0145472	0.00366097
p2–p3	*	0.07425	0.00366097
p2–p4	*	0.0161917	0.00366097
p3–p4	*	−0.0580583	0.00366097

* Asterisk denotes a statistically significant difference.

**Table 15:** Means and 95.0 percent LSD intervals for DSC by epochs.

E	Cases	LS Mean	LS Sigma	Homogeneous Groups
100	96	0.65554	0.00113823	A
200	96	0.665257	0.00113823	B
325	96	0.674076	0.00113823	C

**Table 16:** Contrast comparison by epoch.

Contrast	Significant	Difference	+/− Limits
100–200	*	−0.00971771	0.00317049
100–325	*	−0.0185365	0.00317049
200–325	*	−0.00881875	0.00317049

* Asterisk denotes a statistically significant difference.

**Table 17:** Two-way analysis of variance for time (s)—Type III sum of squares.

Source	Sum of Squares	Df	Mean Square	F-Ratio	*p*-Value
**Main Effects**					
A: Dataset	3173.39	1	3173.39	0.14	0.7045
B: Model	$1.73038\times 10^{9}$	1	$1.73038\times 10^{9}$	78,601.33	**0.0000**
C: Preprocessing	$5.56373\times 10^{6}$	3	$1.85458\times 10^{6}$	84.24	**0.0000**
D: Polynomial Scheduler	14,121.0	2	7060.52	0.32	0.7259
E: Epochs	$3.54111\times 10^{9}$	2	$1.77056\times 10^{9}$	80,426.45	**0.0000**
**Interactions**					
AB	53,901.4	1	53,901.4	2.45	0.1189
AC	11,441.6	3	3813.86	0.17	0.9144
AD	99,782.6	2	49,891.3	2.27	0.1058
AE	27,159.5	2	13,579.7	0.62	0.5405
BC	$5.11692\times 10^{6}$	3	$1.70564\times 10^{6}$	77.48	**0.0000**
BD	105,749.0	2	52,874.3	2.40	0.0927
BE	$3.24992\times 10^{8}$	2	$1.62496\times 10^{8}$	7381.28	**0.0000**
CD	24,929.3	6	4154.89	0.19	0.9798
CE	834,961.0	6	139,160.2	6.32	**0.0000**
DE	21,317.5	4	5329.38	0.24	0.9143
**Residual**	$5.43761\times 10^{6}$	247	22,014.6		
**Total (Corrected)**	$5.61379\times 10^{9}$	287			

**Table 18:** Means and 95.0 percent LSD intervals for time (s) by model (M).

M	Count	LS Mean	LS Sigma	Homogeneous Group
m1	144	5655.74	12.3644	A
m2	144	10,558.1	12.3644	B

**Table 19:** Contrast comparison by model.

Contrast	Significant	Difference	+/− Limits
m1–m2	*	−4902.35	34.4406

* Asterisk denotes a statistically significant difference.

**Table 20:** Means and 95.0 percent LSD intervals for time (s) by preprocessing (P).

P	Count	LS Mean	LS Sigma	Homogeneous Groups
p3	72	7875.42	17.4859	A
p1	72	8150.99	17.4859	B
p2	72	8154.9	17.4859	B
p4	72	8246.33	17.4859	C

**Table 21:** Contrast comparison by preprocessing.

Contrast	Significant	Difference	+/− Limits
p1–p2		−3.91667	48.7064
p1–p3	*	275.569	48.7064
p1–p4	*	−95.3472	48.7064
p2–p3	*	279.486	48.7064
p2–p4	*	−91.4306	48.7064
p3–p4	*	−370.917	48.7064

* Asterisk denotes a statistically significant difference.

## Data Availability

The preprocessed dataset is available at https://www.kaggle.com/datasets/alejf97/unitochest-preprocessed. Code is available at https://github.com/ajf97/Statistical-Analysis-nnUNet. The original data can be found using the information from reference [24] in the following link: https://doi.org/10.5281/zenodo.5797912.
